# Psychological Factors in Dental Patient Care: Odontophobia

**DOI:** 10.3390/medicina55100678

**Published:** 2019-10-08

**Authors:** Rosa De Stefano

**Affiliations:** Department of Biomedical and Dental Sciences, Morphological and Functional Images, University of Messina, 98100 Messina, Italy; rsdestefano@libero.it

**Keywords:** dental anxiety, dentistry, psychological, behavior and behavior mechanisms, anxiety, hypnosis, patient care management

## Abstract

Dentistry and oral health are at the heart of the systemic health of humans. Often this branch of medicine is underestimated either due to socioeconomic reasons or due to fear. In fact, in dentistry, there is often a widespread condition of odontophobia among patients. A clinician’s knowledge of this condition, and an accompanying understanding of how to successfully manage it, is surely one of the first steps to gaining a patient’s trust and maintaining his or her patronage. Being able to manage a dental phobic patient in the best way is the key to successful therapy. Psychological techniques often have to work alongside dentistry in managing these patients. A future perspective concerns precisely the implementation of non-invasive practices such as hypnosis in the management of the latter.

Since 1960, despite the continuous technological evolution, fear not only has shown no hint of diminishing within the world population, but rather has been on the rise. Clinicians and researchers have shown a growing interest in this topic over the past four decades, and have increasingly sought to deepen their understanding of its many facets. The problem of dental phobia (or odontophobia) is closely related to the dentist: it is therefore more important than ever that a dentist possesses, among his or her other skills, knowledge of how to identify it and an understanding of how to manage it in the most appropriate way. Odontophobia has been recognized by the World Health Organization (WHO) as a real disease. According to WHO estimates, it is believed to affect around 15–20% of the population [1].

For people who suffer from this pathology, the dentist is, for different reasons, a real nightmare. These patients are often people who cannot cope with the idea of having a dentist’s session in the face of enormous self-control [1]. Odontophobics are normally inclined to continually postpone treatment, clinging to pharmacological therapies (antibiotics and/or painkillers) which, postponing the solution of the problem, produce a serious worsening of the initial picture with a progressive loss of mastication and a consequent aesthetic compromise [2]. It is true that the dentist intervenes in a particular area of the body, the mouth. A reason that explains the fear of the dentist is given by the peculiarity of the instruments used that undoubtedly can represent anxiety-provoking stimuli, such as the anesthesia syringe [3], the noisy drill, a low speed micromotor, or an ultrasonic scaler that produces a noise [4]. The most statistically stressful experiences are tooth extractions and surgery under local anesthesia [5].

During dental care, the patient is alert and aware of what is happening around him or her. The fear of the dentist could decisively block a patient from receiving dental care—a terrified person may avoid undergoing even simple dental cleaning. These reactions could cause serious problems to oral health and make patients fall into a real self-destructive spiral, where the pain will increase the fear of the dentist, while the situation of the mouth will be compromised, causing a marked worsening of the clinical condition, with the onset of gingivitis, periodontitis, and halitosis [6]. Often these fears are not taken seriously by family and friends who, deriding the subject, may cause the subject to experience real social isolation [7,8]. The dental phobic patient is often not collaborative, even refusing entry into a dental office, instead using painkillers and analgesics in order to avoid undergoing dental treatment. In these cases, any form of treatment to reduce the patient’s anxiety may be considered ineffective, and sedation could be the only solution to obtain results that can effectively treat the patient in a single session [9].

Methods used to overcome odontophobia are different, and they could range from the simple approach of the dentist, who can be friendly and accommodating, or may even include the use of different hypnosis techniques on the patients [10]. Among the techniques related to the clinician’s approach, certain devices such as audiovisual devices or virtual reality devices [11,12,13] that can relax and distract the patient with forms of entertainment during the medical session must certainly be mentioned, or even digital local anesthesia techniques. Some clinical trials also report the effects of some aids, such as art, music, or even the presence of domestic animals [11,14,15,16]. Furthermore, methods of conscious sedation and deep sedation are recognized, with the use of oral, intravenous, or gaseous drugs [17,18].

The best way to achieve this goal is to offer each patient, while already in the waiting room, a cognitive test with questions aimed at the fear he or she feels towards the dentist. The most widespread test at the international level is the Dental Anxiety Scale, designed by Corah et al. in 1978 [19]. This test, according to the most recent literature, is simple and considered to be the best in terms of internal consistency and test–retest reliability [20,21].

Three classes of dental phobia can be recognized:

(1) Mild odontophobia, also called “dental anxiety”, is the most frequent among the population;
(2) Moderate odontophobia, called “dental fear”;
(3) Severe dental phobia, the real “dental phobia”, decidedly rarer and more difficult to manage by the dentist.

If the Dental Anxiety test reveals a condition of dental fear, the dentist will have to realize that the overexposed techniques of behavior will probably not be sufficient, and will instead have to resort to a whole range of sedative support techniques. These techniques include oral pharmacological anxiolysis, inhalation or intravenous conscious sedation, and clinical hypnosis.

Pharmacological anxiolytic treatment is certainly one of the more affordable techniques for a dentist who does not have equipment for conscious inhalation sedation or does not have a wealth of direct hypnotic techniques. The most commonly used drugs are short-lived benzodiazepines taken orally, which have the considerable advantages of practicality, ease of administration, and absolute safety [22,23].

Intravenous conscious sedation consists of the administration of sedative drugs, mainly benzodiazepines, by injection and cannulation in the venous pathways. An excellent sedation technique, which has the advantage of not requiring the presence of the anesthesiologist in the clinic, is conscious sedation by inhalation. Finally, if there is a severe dental phobic patient, then elaborate techniques are required with major contraindications.

Deep sedation is “a state of induced depression of the consciousness accompanied by a partial loss of protective reflexes, including the ability to continuously maintain the airways and/or respond adequately to physical stimulation and verbal commands”. The most widely used pharmacological associations are benzodiazepines and barbiturates or benzodiazepines and morphine [18,24].

General anesthesia is “a pharmacologically induced state, temporary and reversible, of unconsciousness, associated with loss of protective reflexes, including the inability to maintain airway patency, without the fundamental functions of the life” [25].

Dentists should send severe dental phobic patients, and in some cases even moderately dental phobic patients, to a psychologist or psychotherapist as soon as possible. It is essential that the patients are helped by a specialist to find the strength to face the fear that afflicts them and to overcome the obstacle affecting their personal health and life in general.

This is the best way to be concretely useful to the patient, as the use of sedative techniques does not allow the patient to change his or her way of reacting to the phobic situation and to perceive the dental reality [1,26,27]. Through the support of a psychologist, the patient will be able to slowly change his or her beliefs, emotional and cognitive reactions, and the automatic thoughts associated with the perception of the phobic stimulus. Finally, the patient will be able to undergo dental sessions without fear, recover his or her health and smile, regain self-confidence, and permanently escape the yoke of dental phobia.
